# Diagnostic workup for endometrioid borderline ovarian tumors (eBOT) requires histopathological evaluation of the uterus

**DOI:** 10.1186/s13048-021-00839-4

**Published:** 2021-07-07

**Authors:** Juliane Reichenbach, Elisa Schmoeckel, Sven Mahner, Fabian Trillsch

**Affiliations:** 1 Department of Obstetrics and Gynecology, University Hospital, Ludwig-Maximilian-University of Munich, Marchioninistrasse 15, 81377 Munich, Germany; 2 Department of Pathology, Ludwig-Maximilian-University of Munich, Thalkirchner Strasse 36, 80337 Munich, Germany

**Keywords:** Endometrioid borderline ovarian tumor, Uterine curettage, Fertility preservation, Endometrial disorders, Ovarian metastases, Endometrial cancer, Cervical cancer, Reproductive oncology, Fertility-sparing surgery

## Abstract

**Background:**

For young borderline ovarian tumor (BOT) patients, preservation of the uterus was incorporated as an accepted option into treatment guidelines. For the endometrioid subtype (eBOT) however, adequate histological evaluation is challenging and might be associated with synchronous endometrial disorders or misinterpreted as spread from uterine primaries.

**Case presentation:**

We report the cases of two young patients with eBOT who underwent treatment according to current guidelines. In both cases, unexpected findings of invasive uterine carcinomas were established in final histopathological evaluation.

**Conclusions:**

This constellation highlights the challenging diagnostic workup of BOT and underlines that uterine curettage is indispensable for eBOT to exclude uterine primary tumors when fertility preservation is planned. Accordingly, we suggest to include this procedure into recommendations for diagnostic workup and to state the potential risk in treatment guidelines.

## Background

Borderline ovarian tumors (BOT) are epithelial tumors described as an intermediate form between malignant and benign neoplasms with enhanced atypical cellular proliferation but without invasive growth pattern [1]. In accordance with ovarian cancer, six different histological subtypes are distinguished: serous and mucinous histology accounting for approximately 95% of all BOT, and the infrequent forms of endometrioid, clear cell, seromucinous and Brenner Borderline tumors [1]. Endometrioid histology (eBOT) represents approximately 2–3% of all BOT. Histopathologically, eBOT consist of atypical endometrioid glands or cysts located in fibrous stroma without stroma invasion and originate from either ovarian epithelial cells or endometriosis [1]. Due to its low incidence, histopathological evaluation is challenging and it is difficult to give evidence based recommendations for treatment and follow-up.

According to ovarian cancer, comprehensive surgical staging for BOT in general includes bilateral salpingoophorectomy, exploration of the whole abdominal cavity with peritoneal washings, an omentectomy and multiple peritoneal biopsies. Due to low risk for tumor involvement, hysterectomy can be omitted following informed consent to reduce operative morbidity if this procedure is not necessary for complete cytoreduction [2]. Minimally invasive surgical approach has been accepted to be safe for early stage disease with small volume and in the absence of extensive peritoneal implants [3, 4].

Given that a considerable number of BOT occur in women of reproductive age, discussion of fertility conservation is important and preserving at least parts of one ovary and the uterus has become widely accepted for patients with desire to get pregnant [3, 4]. According to the low risk for invasive recurrence of 0,5% after fertility sparing surgery, current ESGO recommendations discuss that preservation of the uterus should be considered even if intact ovarian tissue cannot be preserved [5]. So far, biological, pathological, and molecular behavior as well as different therapeutic approaches according to the histological subtype are not specifically addressed in most treatment recommendations [3]. Albeit not frequently observed, simultaneous occurrence of endometrial disorders in the uterus has been reported [1, 6]. In this context, the following two clinical cases will emphasize the importance of an adequate diagnostic workup of patients with suspected eBOT to exclude an invasive extraovarian primaries.

## Case presentation 1

**Fig. 1:**
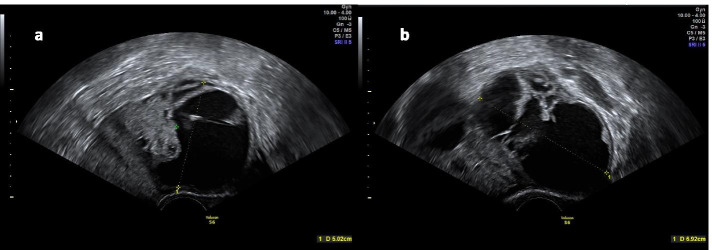
**a**, **b** Presentation at vaginal ultrasound. Bilateral adnexal masses with both cystic and solid aspects as well as a remarkable hyperperfusion were noted at vaginal ultrasound. (**a**) left ovary, (**b**) right ovary

**Fig. 2:**
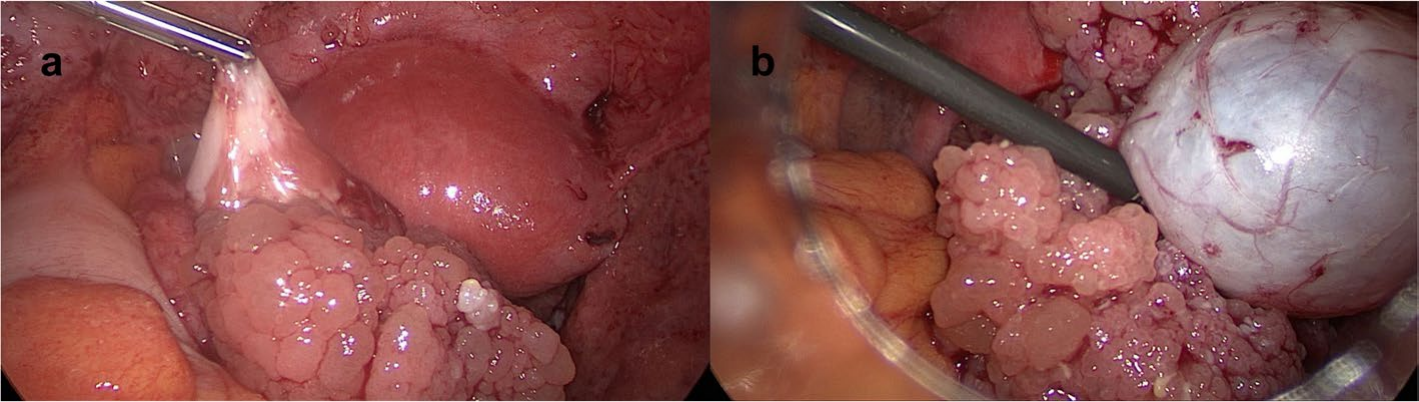
**a**, **b** Intraoperative findings*.* Unlike the suspected bilateral tuboovarian abscesses, ruptured ovarian tumors with up to 8 cm sized cauliflower-like masses comprised of small cysts foremost of the right, but also of the left ovary were detected during emergency laparoscopy. (**a**) left ovary, (**b**) right ovary

A 36-year old nulliparous female patient presented to our emergency department with acute lower abdominal pain, general discomfort, and elevated inflammatory markers. Her past medical history included obesity with a body-mass-index of 40, superficial vein thrombosis of the upper extremity and achilles tendon rupture. No bleeding abnormalities were reported. Cervical cancer screening including PAP smear was regularly performed and inconspicuous, most recently less than one year ago. On physical examination the patient presented significant rebound tenderness and involuntary guarding of the lower abdomen with a positive Blumberg sign. Speculum examination showed normal external female genitalia, normal vaginal epithelium, no abnormal discharge and a non-suspicious cervix. Significant cervical motion tenderness was observed during vaginal palpation. On vaginal ultrasound, the anteverted and anteflexed uterus showed no suspicious lesions. Bilateral adnexal masses with both cystic and solid aspects, remarkable hyperperfusion as well as free intraabdominal fluid were noted (Fig. 1a, b), suspicious for tubo-ovarian abscesses. During emergency laparoscopy, ruptured ovarian tumors with up to 8 cm sized cauliflower-like masses comprised of small cysts foremost of the right, but also of the left ovary were detected (Fig. 2a, b) and an enucleation of the cystic structures was performed.

**Fig. 3:**
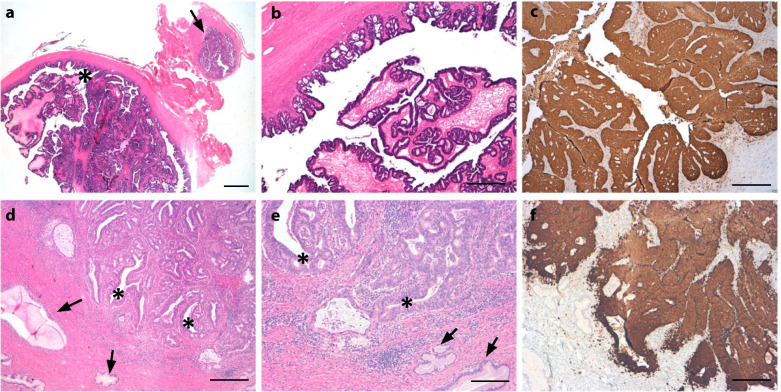
**a**-**f** Endometrioid adenocarcinoma of the cervix uteri with ovarian metastasis. Ovary (**a**-**b**): Cystic enlarged ovary showing extensive papillary and cribriform proliferations of an endometrioid adenocarcinoma (asterisk); Adjacent is a normal tube (**a**, arrow). Scale bars: **a** = 3 mm, **b** = 500 μm. Cervix (**d**-**e**): Adenocarcinoma with cribriform growth pattern (asterisk) in the endocervix next to normal endocervical glands (arrows). Scale bars: **d** = 500 μm, **e** = 200 μm. Consistent with HPV high-risk association the tumor shows strong immunohistochemical expression of p16 in the ovary (**b**) and in the endocervix (**f**). Scale bars: b and f = 500 μm

On histopathological examination, a neoplasm with adeno-papillary growth of the inner female genital tract was described and the diagnosis of an eBOT with microinvasion of up to 1 mm was established. Following thorough postoperative discussion of the treatment options and their clinical impact, the patient decided against a fertility preserving approach favoring radical surgery including hysterectomy. Subsequently, laparoscopic staging was performed with hysterectomy, bilateral salpingo-oophorectomy, infracolic omentectomy and extensive peritoneal staging. In final histopathological results, an unexpected, HPV-high risk associated, intracervical endometrioid adenocarcinoma of the cervix uteri with a transversal diameter of 12 mm was diagnosed so that this was attributed as the primary tumor with ovarian metastases (Fig. 3a-f). Consistent with HPV high-risk association, the tumor showed strong immunohistochemical expression of p16 in the ovary and in the endocervix. CT scan of the thorax and abdomen showed no other distant metastases so that a subsequent laparoscopic lymph node staging was carried out to excluded tumor-infiltrated pelvic or para-aortic lymph nodes. Accordingly, final histopathological assessment led to a tumor stage of pT1b1 with ovarian metastases, pN0, L0, V0, Pn0, G2, R0, FIGO IB1. As individual decision making, an extended adjuvant treatment was applied consisting of chemoradiation followed by chemotherapy with four cycles paclitaxel and carboplatin according to the protocol of the currently recruiting OUTBACK trial (NCT01414608).

## Case presentation 2

**Fig. 4:**
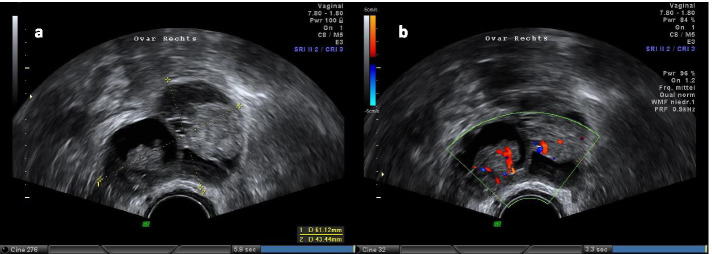
**a**, **b** Sonographic presentation at first consultation. At vaginal ultrasound, the right ovary presented highly suspicious, consisting of cystic and solid aspects. **a** right sided ovarian tumor, **b** doppler ultrasonography of the ovarian tumor

Before presentation to our hospital, the 27-year old nulliparous female, was diagnosed with a left-sided endometrioid BOT at an external institution. She had already undergone fertility-sparing surgery with adequate staging procedures including left-sided salpingo-oophorectomy and omentectomy by open surgery. External histopathological findings revealed a single peritoneal implant in the left paracolic gutter. Three months later she presented for first consultation to our department with a highly suspicious contralateral ovary in vaginal ultrasound (Fig. 4a, b). On physical examination, the patient presented with no acute distress and a soft, nontender, nondistended abdomen. She denied any abnormal vaginal bleeding. Pelvic examination was unremarkable as well as cervical cytology. Vaginal ultrasound revealed a 7 mm thickness endometrium of with slightly inhomogeneity but without any suspicious lesions. The right ovary presented highly suspicious for malignancy consisting of cystic and solid aspects with abnormal perfusion.

**Fig. 5:**
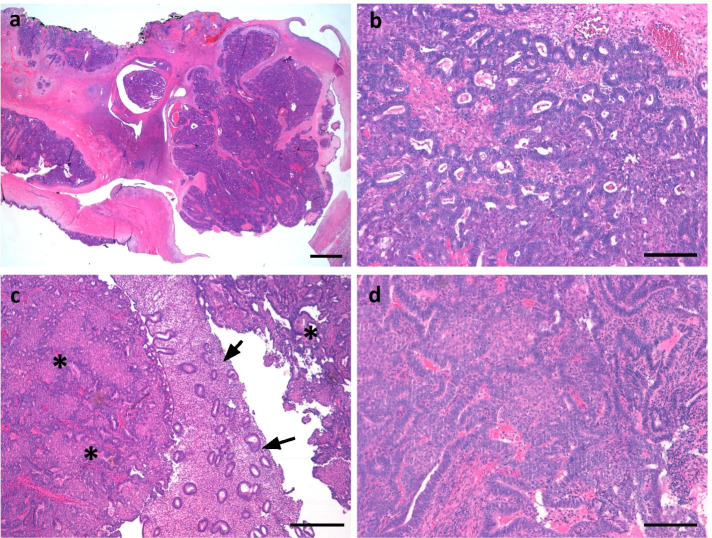
**a**-**d** Synchronous endometrioid adenocarcinoma of the ovary and the uterine corpus. Ovary (**a**-**b**): Endometrioid adenocarcinoma with nodular proliferation and cribriform growth pattern within the ovary. Scale bars: **a** = 3 mm, **b** = 200 μm. Uterine corpus (**c**-**d**): Endometrioid adenocarcinoma in the uterine curettage (asterisk) next regular proliferative phase endometrium (arrows). Scale bars: **c** = 500 μm, **d** = 200 μm

According to her explicit request for fertility preservation, we performed a re-laparotomy with salpingo-oophorectomy of the right side and cryoconservation of healthy appearing ovarian tissue. To increase oncological safety in this constellation, hysteroscopy and curettage was additionally performed. Pathological evaluation revealed a progression of the previously diagnosed eBOT to a well-differentiated endometrioid ovarian carcinoma limited to the ovary and sized 25 mm, positive peritoneal washings and a corresponding FIGO stage IC3 with accompanying superficial endometriosis in peritoneal biopsies. Unexpectedly, uterine curettage revealed an endometrioid endometrial cancer (Fig. 5 a-d). As a consequence, completion surgery consisting of a total abdominal hysterectomy, pelvic and para-aortic lymphadenectomy was performed. No further peritoneal lesions were noted. Histopathological examination diagnosed a moderately differentiated endometrioid endometrial cancer without extrauterine tumor growth with a corresponding FIGO stage IA (pT1a pN0 (0/65) L0 V0 Pn0 G2 R0). While both carcinomas of the ovary and of the endometrium displayed similar histomorphological and immunohistochemical characteristics, the possibility of an ovarian dissemination of the endometrial carcinoma could not be ruled out at this point. However, due to the limited extent of the endometrial cancer and the patient´s history of eBOT, it appeared more likely that two independent carcinomas developed synchronously. No mismatch repair deficiency was detected. Finally, an adjuvant mono chemotherapy consisting of six cycles carboplatin was recommended.

## Discussion and conclusions

Almost one third of all patients diagnosed with BOT are aged under 40 years and the preservation of fertility plays an important role in therapeutic decisions [7]. Fertility-sparing surgical approach—defined as the conservation of the uterus and at least parts of one ovary – combined with a proper surgical staging has become therapeutic standard for the management of BOT in young patients over the past years [4, 5].

BOT represent a histopathologically heterogenous group of ovarian masses that are both, from clinical and subjective diagnostic criteria, difficult to determine [1]. In vaginal ultrasound, only one to two thirds of all cases are adequately diagnosed prior to surgery [8]. Definite diagnosis necessitates histopathological evaluation but frozen section analysis serves as important decision-making tool for further intraoperative procedures in this context [5].

eBOT in particular, represent a rare subtype of BOT challenging to be histopathologically distinguished from metastases of gastrointestinal, endocervical or endometrial adenocarcinomas as they show comparable immunohistochemical characteristics [1]. Previous studies reported on up to half of all cases of patients with eBOT having concomitant disorders of the endometrium and occasionally even synchronous endometrioid adenocarcinoma of the uterus, especially in younger and nulliparous patients [9, 10]. Endometriosis cysts, endometrioid adenofibroma and, in particular, deep infiltrating endometriosis with epithelial atypia are frequently associated with eBOT and appear as possible precursor lesions for the development of eBOT which then has potential to further progress to low-grade endometrioid carcinoma. While the differentiation between eBOT and endometrioid adenocarcinoma is not always straightforward, similar and foremost architectural criteria can be applied as to determine atypical hyperplasia from well-differentiated endometrioid adenocarcinoma of the uterine corpus [5, 10, 11]. In the rare case of co-occurrence of endometrioid cancer of the ovary and endometrium—which is reported in less than 3%—the clinical management and the differentiation between two independent cancers and an advanced staged disease is often challenging [12]. As synchronous endometrial and ovarian tumors do not necessarily impair prognosis and clinical outcome due to younger age, earlier stage and lower grade at primary diagnosis [13, 14], independent clinical behavior rather than metastatic dissemination are hypothesized [15]. However, a clonal relationship between synchronous endometrial and ovarian cancers was detected by targeted and whole exome gene sequencing [15, 16], proposing a process of microenvironmental dissemination without the habit to form distant metastases. Similarly, genetical relationship was found in benign lesions and cancers such as synchronous endometriosis and endometrioid ovarian cancers [17], possibly indicating this behavior also for borderline ovarian tumors [16].

Although a conservative surgical approach of borderline ovarian tumors is associated with higher rates of recurrence in remaining ovarian tissue [18], fertility sparing surgery is considered to be oncologically safe as these recurrences are unlikely to undergo malignant transformation, estimated at only 0.5% and still 2% for advanced disease [3, 4]. Whether the histological subtype of the BOT should be taken into consideration for the surgical management, is still subject of discussion [3, 5]. Due to its low incidence, there is not much data on the oncological safety of fertility sparing surgery in patients diagnosed with eBOT, but previous studies have reported that invasive recurrences of eBOT may occur [6, 9]. While different research groups highlight the importance of uterine curettage and the need of an adequate follow-up in case of a fertility sparing therapeutic approach [1, 9], most international guidelines and treatment recommendations do not specifically address the importance to exclude an extraovarian primary in case of eBOT diagnosis when a fertility conserving approach is envisaged [5, 19–21].

Both presented cases in this report underwent surgical treatment after initial histologic diagnosis of an eBOT and were confronted with unexpected findings of invasive carcinomas of the uterus in histopathological evaluation. In the first case, dissemination of an endometrioid adenocarcinoma of the cervix uteri to the ovaries was initially misinterpreted as a bilateral eBOT at primary diagnosis. The second case was diagnosed with a contralateral invasive recurrence of an eBOT progressed to a well-differentiated endometrioid ovarian carcinoma and a synchronous endometrioid endometrial cancer in uterine curettage. Whether these two occurred as two independent synchronous carcinomas or whether there was an ovarian dissemination from the uterus could not be finally attributed.

Nonetheless, both cases emphasize the importance of uterine diagnostics to exclude primary uterine neoplasm in case of eBOT diagnosis with an envisaged fertility sparing approach. Therefore, we suggest to perform uterine curettage when the uterus should be preserved to prevent possible underdiagnoses of a malignant primary tumor. This could result in wrong treatment decisions with a consecutive undertreatment from an oncologic perspective. Especially in patients designated to get pregnant, this might worsen the prognosis as there is a considerable risk for a delayed diagnosis of ovarian or uterine pathologies. Therefore, the requirement of uterine curettage as part of diagnostic workup to exclude endometrial pathology in case of eBOT with envisaged fertility preservation needs to be stated more prominent in treatment guidelines for young patients with BOT.

## Data Availability

Not applicable.

## Abbreviations

CT: Computertomography
BOT: Borderline ovarian tumor
eBOT: Endometrioid borderline ovarian tumor
ESGO: European Society of Gynaecological Oncology
FIGO: Fédération Internationale de Gynécologie et d'Obstétrique
HPV: Human papillomavirus
